# A step‐by‐step protocol for isolation of murine nucleus pulposus cells

**DOI:** 10.1002/jsp2.1073

**Published:** 2019-12-19

**Authors:** Andrew Bratsman, Greig Couasnay, Florent Elefteriou

**Affiliations:** ^1^ Department of Orthopedic Surgery Baylor College of Medicine Houston Texas; ^2^ Department of Molecular and Human Genetics Baylor College of Medicine Houston Texas

**Keywords:** aging, gene expression, intervertebral disc degeneration, nucleus pulposus

## Abstract

The intervertebral disc (IVD) is composed of three separate tissues with distinct origins and properties. Elucidating changes occurring in these tissues in response to injury or age is paramount to identify new therapies to better manage disc and spine degenerative conditions, including low back pain. Despite their small size and different mechanical load pattern compared to higher species, the use of mouse models represents a cost‐effective and powerful approach to better understand the formation, maintenance, and degeneration of the IVD. However, the isolation of the different compartments of the IVD is complicated by their diminutive size. Here, we describe a simple, step‐by‐step protocol for the isolation of the nucleus pulposus (NP) tissues that can then be processed for further analyses. Analysis from mouse NP tissues shows sufficient quantities of RNAs, purity of the NP fraction, and overall RNA quality for gene expression studies, and reveals no increase in expression of disc degeneration markers, including *TNFa*, *IL1b*, and *Mmp1* up to 15 months of age in C57BL6 wildtype mice.

## 1 INTRODUCTION

The intervertebral disc (IVD) plays an important role in diffusing the various mechanical forces exerted on the spine during movement. In humans, degeneration of the IVD is associated with the onset of neck and lower back pain, a disabling, widespread and costly disease.^1^, ^2^ IVD degeneration is difficult to treat, with current treatments limited to palliative and surgical options. The fact that regenerative strategies for IVD degeneration have to date been unsuccessful reflects our incomplete understanding of the underlying etiology.^3^ The different cellular composition of the three distinct tissues of the IVD, the inner gelatinous nucleus pulposus (NP), the surrounding fibrous annulus fibrosus (AF), and the cartilage endplates (CEP), further complicates studies of this degenerative disease process.

Rich in water, the NP is crucial in preventing stress concentrations in the IVD by distributing forces evenly across this structure. The NP contains cells with different morphologies but all derived from the notochord^4^, ^5^: large, vacuolated notochordal cells (NC) and smaller chondrocyte‐like cells. Multiple studies support an anabolic and protective effects of NC cells on chondrocyte‐like cells and their disappearance is associated with the onset of degenerative disc disease.^6^, ^7^, ^8^ The collagen‐rich AF surrounds the NP in concentric laminae, providing both tensile strength and elastic rebound after movement or compression. AF cells are fibroblast‐like and still poorly characterized. Differing from the NP, where the transcription factor Brachyury (*Bra*) is highly and specifically expressed, the embryologic origin of AF and CEP cells is the sclerotome,^9^ where Paired‐box1 (*Pax1)* is highly expressed. Lastly, the CEP serves as a transition between the vertebral body and the IVD. It is composed of hyaline cartilage that surrounds chondrocytes morphologically similar to articular chondrocytes, although genetic expression studies have shown differences between the CEP and articular cartilage.^10^

Healthy, nondegenerate human samples are limited in their ease of procurement and quantity. Therefore, animal models have been utilized to better characterize healthy cell behavior and characteristics, disc formation and the degenerative process, despite the recognized morphologic differences between IVDs from small animals and humans.^11^ Studies in larger animals are aided in cell isolation by the gross morphological differences between the gelatinous NP and the fibrocartilaginous AF/CEP—these methods utilize gross dissection and further enzymatic digestion to ensure a high yield of cells.^12^ Mice present an excellent opportunity for studying disc degeneration with the wide availability of transgenic models and low overall costs, but cell isolation from each of the IVD compartments is challenging, as the size of the mouse IVD and number of cells within each IVD compartment are far less compared to larger organisms. There is no consistent method for isolating cells of the different IVD compartments in mice for either cell culture or RNA expression (Table 1). Recently, efforts have focused on cell isolation without the use of enzymes, with concerns that enzymatic digestion can alter RNA and/or cell surface expression profiles, require longer expansion times in culture and necessitate multiple passages that leads to general dedifferentiation.^27^ Due to the typical small NP cell yields in mice, many preclinical studies have also resolved on harvesting cells from multiple discs and pooling their contents, usually pooling discs from the same spine region, as differential composition or gene expression between IVDs from lumbar and sacral regions have been reported.^28^, ^29^, ^30^, ^31^ Lastly, because the NPs function in a unique, hypoxic, and hyperosmotic environment, gene expression is likely to change in response to enzymatic or nonenzymatic extraction due both to the time required for the procedure and the relatively dissimilar environment of cell culture conditions versus tissue. For studies focused on phenotyping IVD tissues, there is a need to prepare RNAs of the different IVD compartments in a consistent, fast, and reproductible fashion, without an extra step of ex vivo culture. Main goals of cell isolation from the mouse IVD are thus threefold: (a) preserve enough cells to allow experimental studies, (b) maintain the gene expression profile of IVD cells and limit variations and artifacts due to retrieval or culture methods, and (c) do so in an accessible and easily reproducible method with dependable results. In this report, we describe a new method to isolate pure NP RNAs from murine lumbar and thoracic IVDs, based on a simple centrifugation step.

**Table 1:** Examples of methods used to prepare mouse IVD tissues (nonexhaustive)

Purpose of study	Separation AF/NP?	Method of dissection	Vertebral level	Enzymatic digestion	Ref.
Cell culture	Mixed, NP/IAF sample	Cut was made through the middle of the AF with a scalpel blade. Exposed NP and IAF were scooped out with a needle	Lumbar	0.01% collagenase	13
Cell culture	Yes	Identified and separated under microscope	Tail	0.1% pronase and 0.2% collagenase type 2	14
Cell culture	No	Microscopy was used to harvest IVD tissue completely from the end plates	Coccygeal	No	15
Cell culture	Yes	The disc was cut and bent, forcing the NP to protrude	Coccygeal	No	16
Gene expression	Yes	Dissected	Lumbar + caudal	No	17
Gene expression	Yes	NP was scraped off with a scalpel. AF was isolated by a blade under the stereo microscope. The CEP was shaved off with a scalpel	Lumbar + coccygeal	No	18
Gene expression	Yes	NP and AF from all lumbar IVD were resected separately	Lumbar	No	19
Gene expression	No	Full tissue examined together	Lumbar	No	20
Gene expression	No	Carefully dissected	Unknown	No	21
Gene expression	No	Separated from all other tissues	Tail/Lumbar	No	22
Gene expression	No	No method listed	Unknown	Unknown	23
Gene expression	No	Isolated by microdissection	Thoracic	No	24
Gene expression	No	NP isolated by microdissection	Lumbar + thoracic	No	25
Gene expression	Yes	NP and AF isolated by microdissection	Lumbar + thoracic	No	26

## 2 MATERIALS AND METHODS

### 2.1 Mice

Mice were euthanized by cervical dislocation under general anesthesia induced by isoflurane inhalation. All procedures were approved by the institutional IACUC committee.

**Figure 1:**
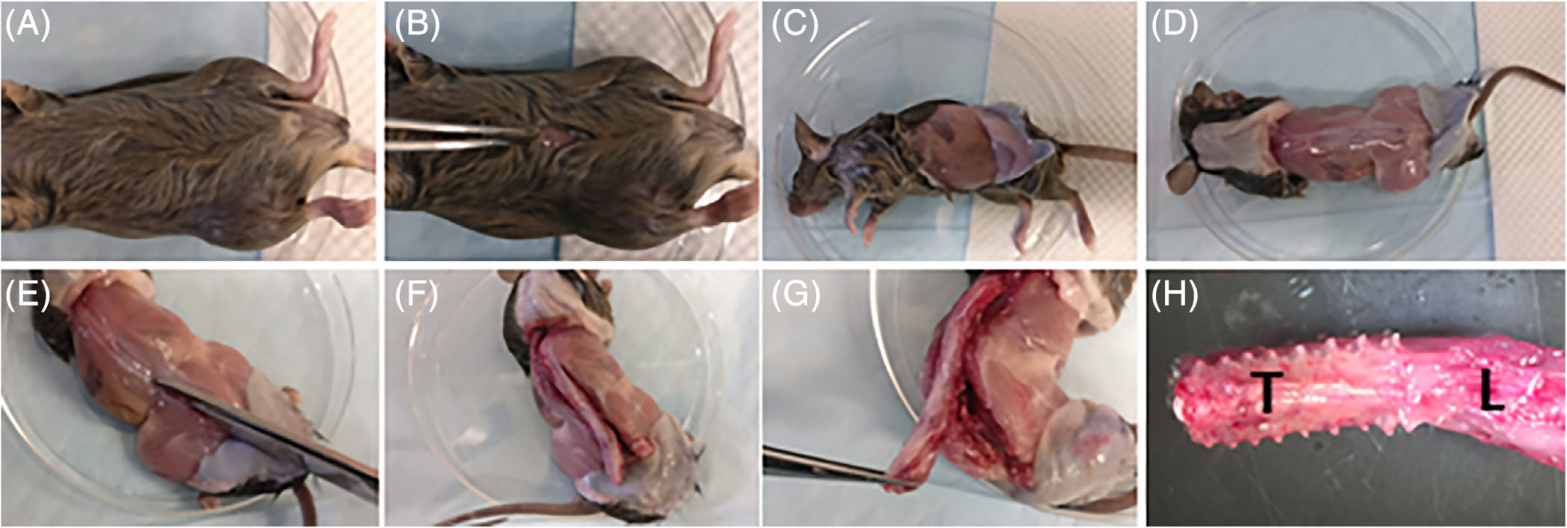
Spinal column isolation. A, Douse the fur with 70% ethanol to prevent contamination and limit spread of hair. Place the mouse on its back. B, An incision is made at level of the umbilicus on the ventral skin. C, A transverse tear is made either manually or with scissors in both directions from the incision to the dorsal spine. D, The pelt is pulled to the base of the tail and to the base of the neck. The spinous processes should now be visible, allowing visualization of the boundaries of the spine. E, With scissors, begin excising the spine from its lateral attachments of muscle, bone, and fascia in a parallel position to the spine. The process is easiest if beginning at the base of the tail and working in a superior direction, ending distal to the region of interest. F, Once one side has been cut, proceed to the opposite side, cutting in the same areas. G, Once the spine has been freed from its lateral attachments, it is still connected both superiorly and inferiorly to the cervical and sacral spine and anteriorly by viscera. Make a transverse cut below the attachment of the femurs, and, pulling the spine away from the body, begin cutting the anterior attachments. Proceed superiorly until the regions of interest have been detached anteriorly, and make another transverse cut through the superior attachment, freeing the spinal column from the body. H, The excised spine. L, lumbar region; T, thoracic region

**Figure 2:**
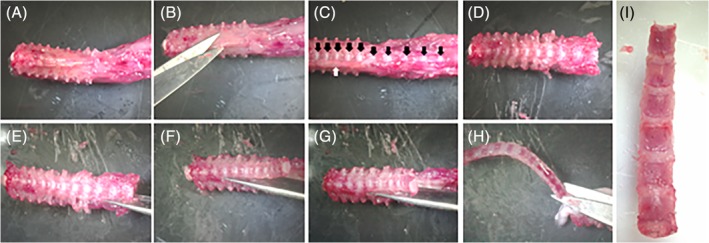
Isolation of the vertebral column from the spine. A, Display of the thoracic and lumbar segments of the spine after spine isolation. B, Using scissors or a scalpel, remove any muscles, fat, or other soft tissues adhering to the anterior part of the vertebral column. C, Removal of the soft tissue exposes the IVDs (black arrows). Tissue continues to be attached on the lateral portions of the IVD (white arrow). D, Continue removing soft tissue from the lateral portions of the vertebral column. This makes the future isolation of the IVD tissue simpler. E, Using scissors, begin cutting through the pedicles, parallel to the vertebral column, to isolate the vertebral column from the remainder of the bony spine. This is best done by inserting one tip of the scissors into the spinal canal and orienting the other tip close to the vertebral body but not on top of it. G, Continue cutting the pedicles on one side of the vertebral column. After that side is cut, proceed to the opposite side. H, Remove any adherent tissues connecting the vertebral column to the remainder of the spine. I, Isolated vertebral column, cleaned of adherent soft tissues

**Figure 3:**
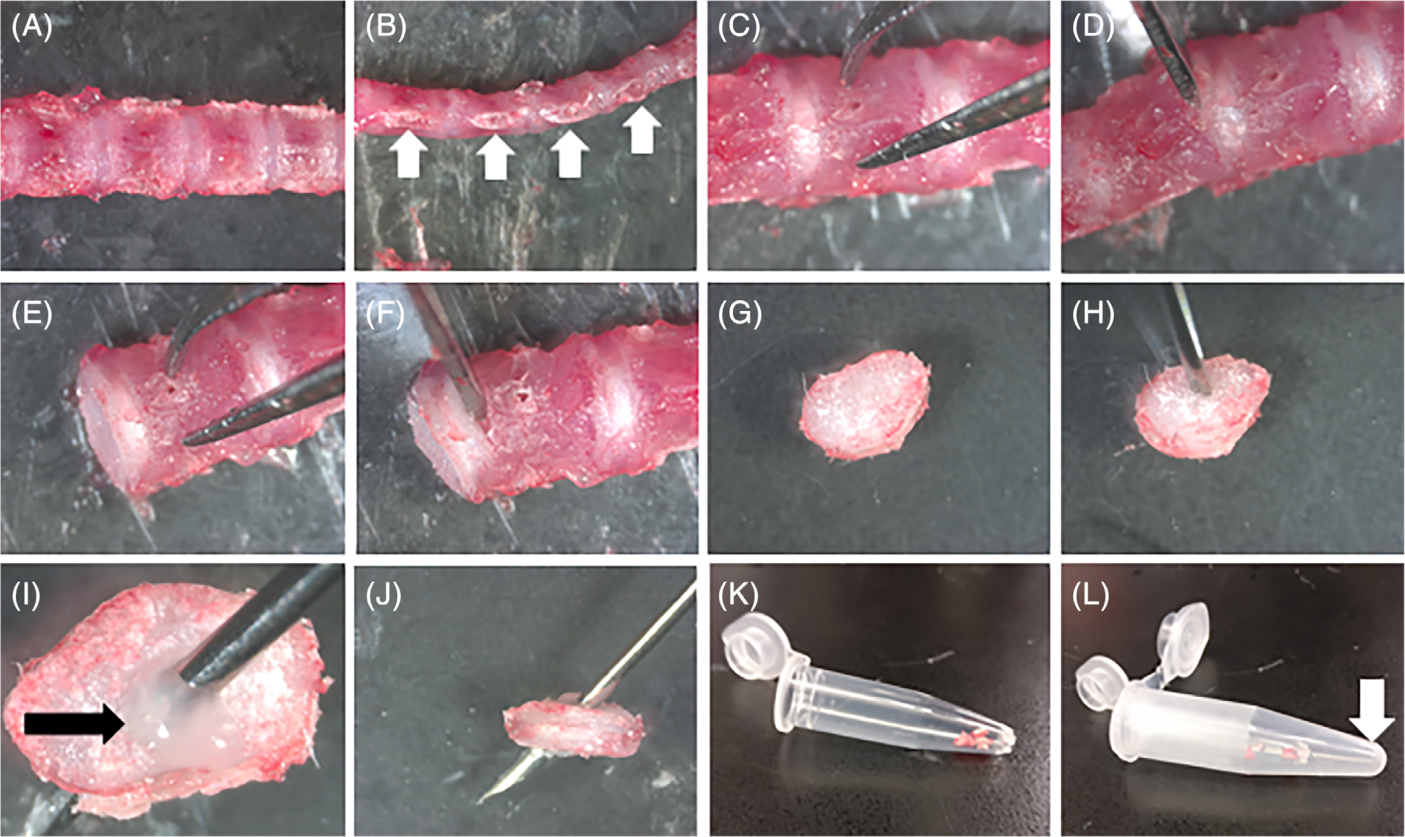
Isolation of the IVD. A, Posterior view of the isolated spine. B, Lateral view of the isolated spine, with the cut pedicles visible (white arrows). C, Proper placement of fine needle tweezers to avoid damage to the IVD. Isolation is simplified by placing the IVD with the posterior portion resting on the iced dissection field and the tweezers making contact on the anterior aspect. D, Proper placement of the scalpel blade on the boundary between the IVD and the superior vertebral body. E, Appearance of the IVD after removal of the superior vertebral body. F, Placement of the scalpel blade to remove the inferior vertebral body. G, Isolated IVD. H, Proper placement of the needle in the superior surface of the IVD. I, Presence of the NP should be confirmed by the appearance of a clear gelatinous NP (black arrow) protruding through the punctured surface. J, The needle is continued through the IVD, puncturing the inferior aspect of the IVD. K, The IVD is deposited in the 0.5 mL tube previously prepared. L, The 0.5 mL tube is placed in a larger 1.5 mL tube to be centrifuged. The NP will collect in the larger tube (white arrow) while the AF/CEP will remain in the original 0.5 mL tube

Detailed step‐by‐step protocol for the isolation of NPs and CEPs/AFs:

1. Place the animal on its back. Douse the skin with 70% ethanol to disinfect and prevent hair from spreading during dissection (Figure 1A).
2. To avoid damaging the spine with too deep of a first incision, grasp and pull the skin away from the abdomen with forceps and make a small incision in the skin into the abdominal space (Figure 1B). Manually remove the pelt from the dorsal spine by either tearing or cutting the skin in the transverse plane and then pulling the two segments up to the base of the neck and below the base of the tail (Figure 1C,D).
3. Isolate the spinal column from the surrounding tissues—using scissors, remove the musculature and long bones, ribs, and pelvis to cut in a parallel direction to the exposed spine from the sacrum and up to the thoracic region (Figure 1E,F). Complete the isolation of the spine by making a transverse cut at the level of the femurs and at the appropriate level in the lumbar/thoracic/cervical region, depending on the region of interest (Figure 1G).
4. Move the excised spinal column onto an iced dissection field under a dissecting microscope (Figure 1H and 2A). The iced dissection field can be prepared by filling the cover of a Petri dish with ice and placing the other half on top, decreasing the temperature of the dissecting surface close to freezing point while avoiding contamination of the dissection field. No buffer needs to be used during the fine dissection.
5. With scissors or a scalpel, remove the fascia and musculature attached to the anterior aspect of the vertebral column (Figure 2B). This will expose the IVDs (Figure 2C, black arrows). Continue to remove the lateral attachments of soft tissues (Figure 2C, white arrow), as doing so will simplify the process of isolating the IVDs from the vertebral column (Figure 2D).
6. Cut through both pedicles to isolate the vertebral body from the other parts of the vertebrae. This can best be accomplished by inserting one blade into the spinal canal and positioning the other blade next to the vertebral body (Figure 2E). After cutting one side free, continue to the other side (Figure 2F,G). Remove any additional attachments connecting the vertebral column to the remaining posterior portion of the vertebrae (Figure 2H). Take care to remove any remaining spinal cord that can contaminate samples. Spinal cord can be visualized as a white column that extends the length of the spine.
7. If there is any extraneous soft tissue still attached to the isolated vertebral column, use scissors or a scalpel to remove (Figure 2I).
8. Under a dissecting microscope, examine the vertebral column, placing it with the posterior side down with the anterior aspect facing you (Figure 3A‐C). Hold the bony vertebral body with the pincers to allow proper stabilization of the specimen (Figure 3C). Isolate IVDs from lumbar and thoracic regions keeping the IVD intact (NP + AF + CEP) by placing the scalpel at the exact boundary of the CEP and the vertebral body and gently pushing down (Figure 3D,E). There will be a small color change between the two structures, and if the scalpel blade is placed correctly the structures will separate with very little force. Holding the adjacent vertebral body with the pincer allows proper control of the specimen. Repeat on the opposite aspect of the IVD (Figure 3F).
  ▲ *Critical step*—Excessive force during CEP separation from the vertebral body will force the NP out of the IVD, limiting the amount of usable sample. Be aware of the force applied to the joint and adjust the location of the blade if slight pressure is unable to separate the CEP from the vertebrae.
9. Place the isolated IVD (Figure 3G) on a portion of the iced dissection field and continue isolation of all other IVDs. If upon examination of the isolated IVD, marrow, soft tissue, or bone is visualized, these elements can be removed by carefully holding the IVD and separating them away from the IVD with a scalpel.
10. With a 28‐gauge needle, pierce the IVD from the superior to the inferior aspects (Figure 3H‐J, if NP is spared during dissection, it should be visible as a gelatinous outpouching, see Figure 3I, black arrow) and place in a previously prepared perforated tube‐within‐a‐tube, where a 0.5 mL microcentrifuge tube is punctured several times at its most inferior portion with a 25‐guage needle and placed within a 1.5 mL microcentrifuge tube (Figure 3K,L). Centrifuge at 12 000 rcf for 3 minutes at 4°C. Collect the NP cell mixture in the 1.5 mL microcentrifuge tube and deposit the AF/CEP tissue remaining in the 0.5 mL microcentrifuge tube into a new 1.5 mL tube.
11. Add TRIzol reagent to NPs immediately and thoroughly mix, proceeding then to RNA isolation or freezing.
  ▲ *Critical step*—Remember that the NP cells are enmeshed in a matrix. To better dissolve the cells in TRIzol, pipet up and down multiple times.
12. Snap freeze AFs/CEPs in liquid nitrogen immediately and proceed to the mechanical disruption of the AF/CEP tissues.

*Mechanical disruption of the AF/CEP tissues*:

1. Place the −80°C frozen tube(s) of AF/CEPs in liquid nitrogen to cool.
2. After cleaning the mortar and pestle with water, 70% ethanol and then RNase decontamination reagent, fill clean mortar and pestle with liquid nitrogen to cool the equipment to below freezing.
3. With liquid nitrogen still present in the mortar, deposit the AF/CEP frozen sample into the liquid nitrogen and proceed to grind the sample to a fine dust.
  ! *Warning*—It is essential that the sample remains below freezing temperature in order to reduce RNA damage.
4. With a cold metal spatula, scrape the fine powder down the walls of the mortar into the bottom as the liquid nitrogen evaporates. Deposit into the original labeled cold microcentrifuge tube and place back in liquid nitrogen.
5. Samples may then be stored at −80°C or processed for RNA extraction.
  ! *Warning*—Mortar and pestle must be cleaned in between each sample to remove previous tissue and prevent cross‐contamination.

*Troubleshooting/General caution*: It is possible to include bone fragments and bone marrow with the IVD if insufficient care is not exercised during dissection. Inclusion of bone marrow into the NP fraction will be visualized after centrifugation as a dark red mass at the bottom of the tube. Younger animals are simpler to readily separate the CEP from the vertebral body.

### 2.2 Sample size

IVDs were isolated from a total 32 male mice (ages 1‐15 months) and 35 female mice (ages 1‐7 months) according to the method described herein. Five lumbar IVDs and seven thoracic IVDs were separated from each animal. IVDs from each region were pooled and analyses were performed separately in thoracic and lumbar IVDs.

### 2.3 RNA extraction

Average time for IVD isolation measured from euthanasia to freezing or Trizol addition was 17 minutes. Centrifuged NP or ground AF/CEP samples were homogenized in 500 μL of TRIzol reagent (Ambion, cat. No. 15596026). Extraction was performed according to manufacturer's directions. Gross RNA integrity was first assessed by gel electrophoresis. Yield and purity were measured by OD_260/280_ with a spectrophotometer (Tecan Infinite M200; Tecan Deutschland GmbH, Crailsheim, Germany). Ten RNA samples with acceptable gross RNA integrity were further assessed for RIN (RNA Integrity Number) with a bioanalyzer (Agilent 2100; Santa Clara).

### 2.4 cDNA synthesis and gene expression analyses

cDNAs were generated using the High‐Capacity cDNA Reverse Transcription Kit (Applied Biosystems, cat. no. 4368813). Real‐time qPCR was performed with 23 ng of starting cDNAs that were mixed with iQ SYBR Green Supermix (Bio‐Rad, cat. no. 1708882) and primers. Primer sequences can be found in Table 2. Cycling conditions consisted of an initial step at 95°C for 3 minutes followed by 40 cycles of a 10‐second period at 95°C and a 45‐second period at 60°C. Specificity of amplification was verified by the presence of a single peak on melting curves. Relative expression was determined using the 2^‐ΔΔCt^ values with *Pnn* used as normalizer. Ct values are shown in Table S1.

**Table 2:** qPCR primer set sequences

Gene	Forward primer (5′→3′)	Reverse primer (5′→3′)
*Pnn*	ACCTGGAAGGGGCAGTCAGTA	ATCATCGTCTTCTGGGTCGCT
*Pax1*	CCGCCTACGAATCGTGGAG	CCCGCAGTTGCCTACTGATG
*Col10a1*	GCATCTCCCAGCACCAGA	CCATGAACCAGGGTCAAGAA
*Bra*	GCTCAAGGAGCTACTAACGAG	CCAGCAAGAAAGAGTACATGGC
*Cd24*	GTTGCACCGTTTCCCGGTAA	CCCCTCTGGTGGTAGCGTTA
*Tnfa*	CCCTCACACTCAGATCATCTTCT	GCTACGACGTGGGCTACAG
*Mmp9*	CTTCTGGCGTGTGAGTTTCC	ACTGCAGGTTGAAGCAAAGA
*Mmp13*	CAGTCTCCGAGGAGAAACTATGAT	GGACTTTGTCAAAAAGAGCTCAG
*Il1b*	GAAATGCCACCTTTTGACAGTG	TGGATGCTCTCATCAGGACAG
*Rankl*	CAGCATCGCTCTGTTCCTGTA	CTGCGTTTTCATGGAGTCTCA
*Rank*	CACTGAGGAGACCACCCAAG	TGGCAGCCACTACTACCACA
*Ibsp*	CAGGGAGGCAGTGACTCTTC	AGTGTGGAAAGTGTGGCGTT
*Cd31*	ACGCTGGTGCTCTATGCAAG	TCAGTTGCTGCCCATTCATCA

### 2.5 Statistical analyses

Statistical tests are specified in the legend of each figure. When data were analyzed by two‐way ANOVA, tissue compartment (NP vs AF/CEP) and age were the main effects (independent variables). Levene's tests were used to confirm that the data met the equal variance assumption. Where the assumption was violated (*Mmp13*), the data were log transformed to correct the violation. Where the age effect was significant (and the tissue compartment by interaction was not), the age effect was further examined with trend analysis, testing for significant linear, quadratic, and cubic trends to determine if and how gene expression changed over time. Significance was defined as *P* < .05.

## 3 RESULTS

**Figure 4:**
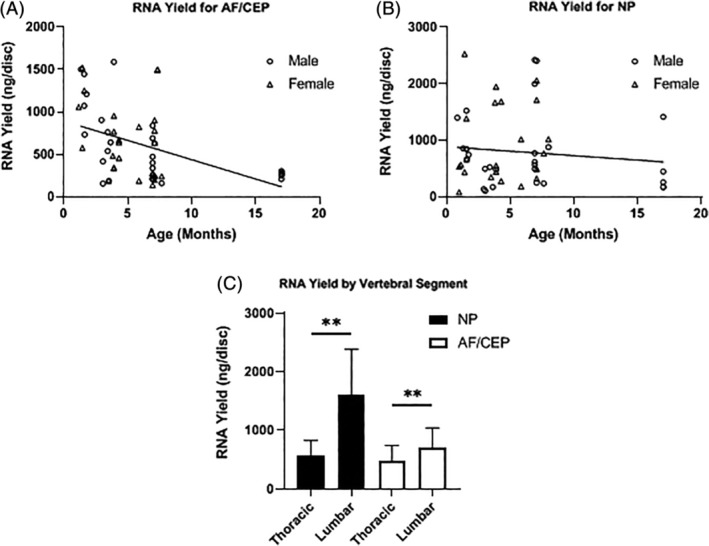
RNA yield by age, gender and IVD structure. A and B, Changing yield of RNA (ng/disc) over time by IVD compartments (n = 5 per age, linear regression for nonzero slope, *P < .05). C, RNA yield by vertebral segment (n = 5 per age, Mann‐Whitney *U* Test, **P < .01)

Average yield of RNA for NP and AF/CEP fractions was 817 and 778 ng/IVD, respectively, when averaging all ages, genders, and spine regions together. Further analysis for average RNA yield per IVD over different ages and per gender revealed variability in the yield of recovered RNA at each age in both NP and AF/CEP fractions, and a general trend toward a reduction in RNA yield in samples from older mice (Figure 4A,B). Significantly more RNA was procured from lumbar NP (*P* = .007) and AF/CEPs (*P* = .006) when compared to thoracic counterparts (Figure 4C). The RIN range was 4.7 to 5.8. There was no correlation between RIN number and age of the mouse.

**Figure 5:**
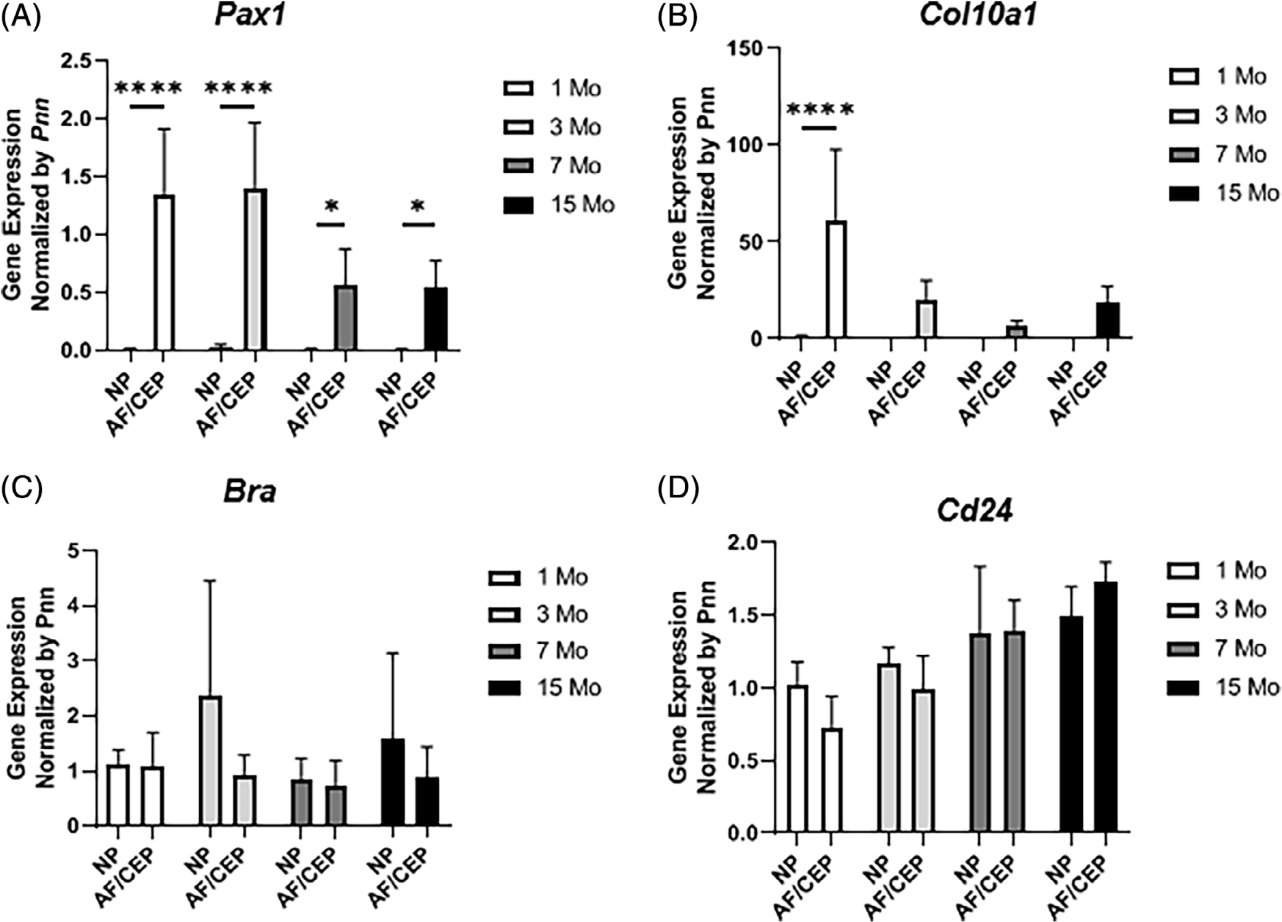
Purity of isolated samples. Gene expression (qPCR) for (A) *Pax1*, (B) *Col10a1*, (C) *Bra*, and (D) *Cd24* in NP vs AF/CEP from 1 to 15 months of age (n = 5 per age, two‐way ANOVA with Sidak's multiple comparison test because of significant interaction for *Pax1* and *Col10a1*. *P < .05, ****P < .0001)

After establishing that this protocol resulted in usable quantities of RNA, samples were examined for purity. The transcription factors *Brachyury* and *Cd24* as well as and *Pax1* and *Col10a1*, were selected as markers of NP and AF/CEP cells, respectively.^4^, ^32^, ^33^ A 73‐ and 69‐fold AF/CEP enrichment vs the NP fractions for *Pax1* and *Col10a1* expression, respectively, across age groups indicated absence or very low AF/CEP RNA contamination in NP samples (Figure 5A,B). In contrast, *Bra* and *Cd24* expression was not different between NP and AF/CEP fractions, thus indicating significant contamination of NP RNA in AF/CEP samples, as these latter genes are not expressed in AF and CEPs (Figure 5C, D).^34^ This result was confirmed histologically on IVD cryosections showing remnants of NP cells following centrifugation (Figure S1A). Compared to AF/CEP fractions, the NP fractions also showed very low (6‐ to 60‐fold weaker) expression of marker genes for mature chondrocyte (*Rankl, Col10a1*), monocytes (*Rank*), osteoblasts (*Ibsp*), and endothelial cells (*Cd31*) (Figure S1B‐E). This method, therefore, demonstrates a good capacity to isolate NP cells from the IVD, but insufficient tissue separation to ensuring an AF/CEP cell mixture free from NP contamination.

**Figure 6:**
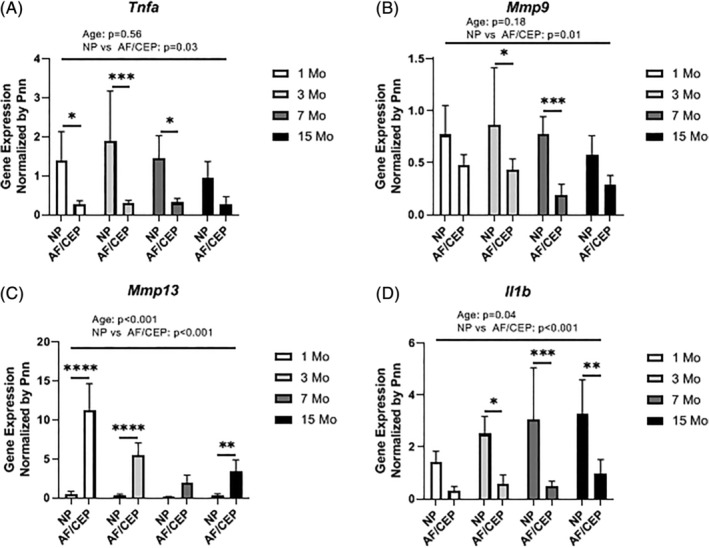
Expression of inflammatory cytokine and metalloproteinase genes. Gene expression (qPCR) for (A) *Tnfa*, (B) *Mmp9*, (C) *Mmp13*, and (D) *Il1b* (n = 5 per age, two‐way ANOVA). Interaction was nonsignificant for all genes tested. Expression differed significantly between NP and AF/CEP for each gene measured (A: *P* = .03, B: *P* = .01, C: *P* < .001, D: *P* < .001). Age was a significant factor for both *Mmp13* and *Il1b* with *P* < .001 and *P* = .04, respectively. Changing expression over time analyzed by orthogonal polynomial contrasts, with a significant linear decrease only for *Mmp13* (*P* < .05), with a nonsignificant linear increase in *Il1b* (*P* = .06). Post‐hoc test with Sidak's multiple comparison test was performed for analysis of NP and AF/CEP expression at each age (*P < .05, **P < .01, ***P < .001, ****P < .0001)

Because prior studies have correlated inflammatory gene expression in mouse IVDs only in the context of disease‐causing treatments or conditions, we took advantage of this longitudinal dataset to measure the expression of inflammatory genes and extracellular remodeling enzymes in NPs and AF/CEPs, with the goal of determining if gene expression changed with age and varied between the two isolated tissue fractions. The expression of tumor necrosis factor (*Tnfa*) and matrix metalloproteinase‐9 (*Mmp9)* was similar with only tissue compartment showing a significant difference (Figure 6A,B). Expression in the NP was significantly greater than in the AF/CEP for both *Tnfa* (F_1,31_ = 5.07, *P* = .03, partial eta‐square = 0.14) and *Mmp9* (F_1,31_ = 7.32, *P* = .01, partial eta‐square = 0.18). Age difference was nonsignificant (alpha = .05). *Mmp13* expression showed no significant age‐by‐tissue compartment interaction, but difference in tissue compartment (F_1,31_ = 392.72, *P* < .01, partial eta square = .92) and age (F_3,31_ = 26.65, *P* < .01, partial eta square = .68) were both significant (Figure 6C). However, in this case, *Mmp13* expression in the AF/CEP was significantly greater than in the NP. Trend analysis of the age alone indicated a significant negative linear effect (t = −1.95, *P* = .05, eta‐square = 0.54), indicating that *Mmp13* expression significantly decreased with aging. The quadratic and cubic trends were not significant (t = 1.4, *P* = .16 and t = 1.11, *P* = .27 respectively). The expression pattern for *Il1b*, a key cytokine implicated in disc degeneration, was similar to that of *Mmp13* with no significant age‐by‐tissue compartment interaction, but significant differences between tissue compartment (F_1,31_ = 46.15, *P* < .01, partial eta‐square = 0.62) and over time (F_3,31_ = 3.11, *P* < .04, partial eta square = 0.25) (Figure 6D). However, like *Tnfa* and *Mmp9*, *Il1b* expression in the NP was significantly greater than the AF/CEP. None of the trends (linear, quadratic, or cubic) were significant (alpha = .05), though Figure 6D suggests a positive linear increase in gene expression over time with a t value approaching significance (t = 1.9, *P* = .06, eta‐square = 0.94).

## 4 DISCUSSION

The method described herein allows multiple and reliable qPCR analyses from lumbar and thoracic mouse IVD compartments and can circumvent the need for pooling IVDs from different spine regions or mice in nonsurgical models. This is important as gene expression may differ between different levels of the spine (cervical vs thoracic vs lumbar vs tail). Likely due to their relatively larger size, lumbar IVDs yielded a greater amount of RNA than thoracic IVDs.

The NP has been implicated as an indicator and driver of IVD degeneration.^35^ The ability to procure a pure fraction of NP tissue allows precise investigation of molecular phenotypes, response to treatments, and the degenerative process separate from the AF/CEP. This prerequisite information can serve as a foundation for future cell‐ and stem cell‐based therapies.

A caveat of the method described herein is the low RIN numbers observed in samples examined for RNA integrity. Although inadequate sample handling or storage may cause RNA degradation, the low RIN values obtained likely result from partial RNA degradation that may occur postmortem during the dissection process, before samples are snap‐frozen or homogenized in guanidine‐containing solutions, as average dissection time to collect both lumbar and thoracic IVDs was over 15 minutes per mouse. One should note, however, that if normalized with a reference gene, RIN does not overtly affect qPCR expression profiles.^36^, ^37^ Thus, although one should always aim at obtaining RNAs with RIN > 5, the method described herein is suitable for most qPCR gene expression analyses. Reducing the time between euthanasia, dissection, and tissue freezing is likely to increase average RIN. Collecting lumbar vertebrae, which yield larger amount of RNAs than other spine regions, is also recommended. Keeping samples at low temperatures during dissection by working on a refrigerated dissection surface is also important. Mechanical disruption of the tissues while submerged in liquid nitrogen follows this principle, as disruption with a bead mill or a mechanical homogenizer exposes the samples to higher temperatures.

By comparing the expression of tissue specific transcription factors, we were able to show that the isolated NP is relatively free of AF/CEP and marrow cell contamination, but the above‐described method is insufficient to result in an AF/CEP fraction free of NP cell contamination, as shown by the detection of NP‐specific gene (*Bra, Cd24*) in AF/CEP fractions and the presence of NP remnants in centrifuged IVDs by histology. Therefore, the gelatinous property of the NP helps this tissue to be extracted by simple centrifugation from the IVDs, but peripheral NP cells remaining within the intervertebral discs during centrifugation contaminate AF/CEP samples.

There have been efforts made to differentiate changes due to the normal physiologic process of aging and the pathologic degeneration seen in degenerative disc disease. For instance, some studies have shown that IL1β and TNFα were key inflammatory cytokines in disc degeneration, released by IVD cells in addition to infiltrating inflammatory cells and able to induce catabolic and anti‐anabolic shifts in bovine and human IVDs.^38^, ^39^, ^40^ We report here no increase in expression of these key inflammatory genes, in the absence of disease‐causing conditions or degeneration‐susceptible gene knockout, up to 15 months of age in the mouse NP. The only significant change in expression with age was a decrease in *Mmp13* expression, which may reflect reduced ECM remodeling. To our knowledge, this is one of the first reports where these genes were examined in the context of aging and nondegenerative state in in vivo mouse IVDs and in different IVD compartments, establishing an important foundation for future studies focused on the correlation between aging and inflammatory gene expression. Our data show that although MRI, radiographic, and histologic evidence of IVD degeneration may occur at earlier ages,^41^, ^42^, ^43^, ^44^ no significant changes in gene expression for inflammatory markers occur up to 15 months of age in mice. These data are in line with a recent study comparing global changes in gene expression during aging in the mouse NPs and AFs by microarray analysis, which did not reveal any genes related to inflammation in the top most upregulated genes.^26^

## 5 CONCLUSION

Mice are important animal models for investigating disease processes and genetic influences in spine development, aging, and degeneration. We present a reliable and reproducible method for isolating murine NP tissue from IVDs. We also show that there is no evidence of inflammation of the mouse NP up to 15 months of age at the gene expression level.

## CONFLICT OF INTEREST

The authors declare no potential conflict of interest.

## AUTHOR CONTRIBUTIONS

A.B., G.C., and F.E. designed experiments. A.B. and G.C. performed and analyzed measurements. A.B., G.C., and F.E. wrote the manuscript. All authors reviewed and approved the manuscript.

## Supporting information


**Supplementary Figure 1 Non‐contamination of isolated samples by marrow components. A**) IVD cryosections from intact and centrifuged lumbar IVDs from 2‐month‐old mice stained by Alcian blue. Picture are representative of 6 IVD isolated from 3 mice. Bar = 50 μm. **B‐E**) Gene expression (qPCR) for **A**) *Rankl*, **B**) *Rank*, **C**) *Ibsp* and **D**) *Cd31* in NP vs AF/CEP in 1‐month‐old mice (n = 5 per age, Mann‐Whitney *U* test, ***: *P* < 0.001, ****: *P* < 0.0001).

**Supplementary Table 1** qPCR CT values.
