# Supplementary figures and images for: A step‐by‐step protocol for isolation of murine nucleus pulposus cells
Source: JOR Spine. 2019 Dec 19;2(4):e1073. doi: 10.1002/jsp2.1073 (PMC6920701; doi:10.1002/jsp2.1073)

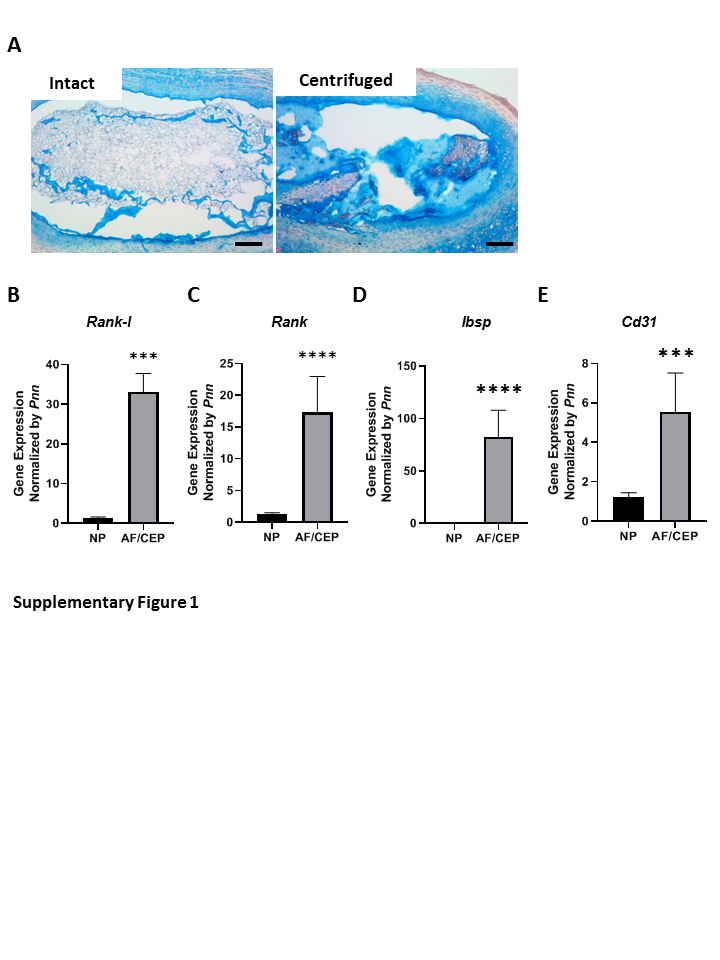

Supplement: Supplementary file 1 — Supplementary Figure 1 Non‐contamination of isolated samples by marrow components. A) IVD cryosections from intact and centrifuged lumbar IVDs from 2‐month‐old mice stained by Alcian blue. Picture are representative of 6 IVD isolated from 3 mice. Bar = 50 μm. B‐E) Gene expression (qPCR) for A) Rankl, B) Rank, C) Ibsp and D) Cd31 in NP vs AF/CEP in 1‐month‐old mice (n = 5 per age, Mann‐Whitney U test, ***: P < 0.001, ****: P < 0.0001). [file JSP2-2-e1073-s001.TIF]

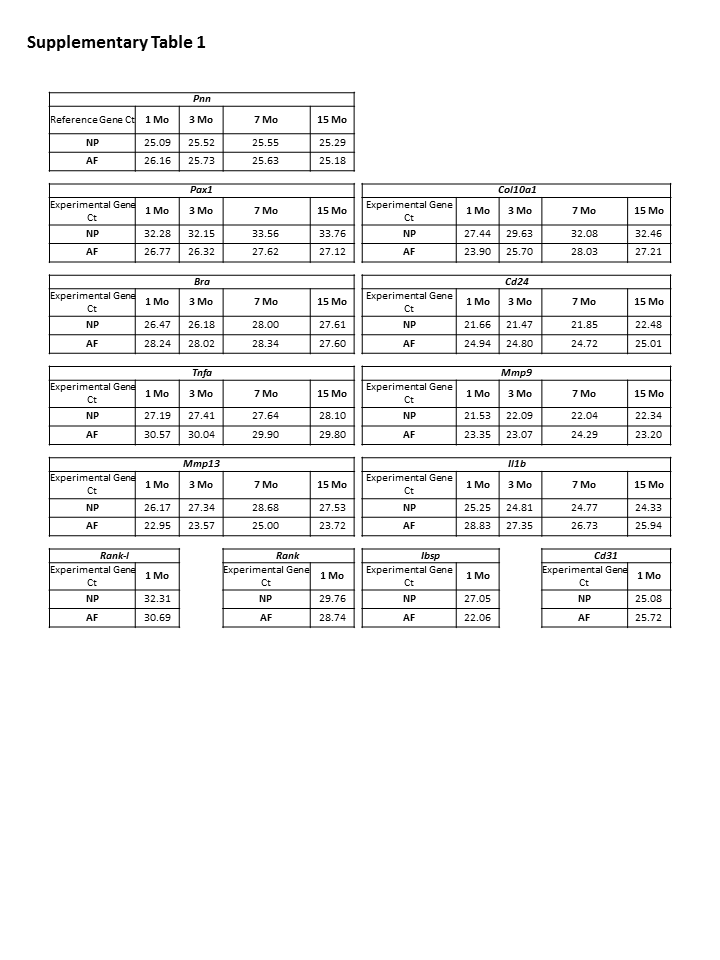

Supplement: Supplementary file 2 — Supplementary Table 1 qPCR CT values. [file JSP2-2-e1073-s002.TIF]
